# Endoscopic Management of Acute Gastrointestinal Bleeding From a Gastric Lipoma: A Case Report, Literature Review, and Treatment Recommendation With Reference to ESGE Guidelines

**DOI:** 10.1155/crgm/6874807

**Published:** 2025-10-03

**Authors:** Ján Csomor, Petr Hříbek, Kateřina Košťálová, Jiří Soukup, Štěpán Suchánek, Petr Urbánek

**Affiliations:** ^1^Department of Internal Medicine, 1^st^ Faculty of Medicine, Charles University and Military University Hospital, Prague, Czech Republic; ^2^Department of Military Internal Medicine and Military Hygiene, Military Faculty of Medicine, University of Defence, Hradec Kralove, Czech Republic; ^3^The Fingerland Department of Pathology, University Hospital Hradec Kralove, Hradec Kralove, Czech Republic; ^4^Department of Pathology, First Faculty of Medicine, Charles University and General University Hospital in Prague, Prague, Czech Republic

**Keywords:** gastrointestinal bleeding, lipoma, subepithelial lesion

## Abstract

**Background:**

Acute gastrointestinal bleeding from a gastric lipoma is a very rare but severe condition, about 50 cases of which have been reported in the worldwide literature at the time of writing. It largely occurs from a lesion larger than 4 cm localized in the antrum of the stomach.

**Objective:**

We report the case of a patient with severe comorbidities presenting with melena and, on examination, a large subepithelial lesion in the gastric antrum, with a small ulceration and visible vessel at the summit. After stabilization and endoscopic hemostasis, the lesion was successfully treated by endoscopic submucosal dissection and a diagnosis of a large gastric antral lipoma was confirmed. We have collected similar case reports published worldwide and made a careful review of the literature and ESGE guidelines; we present these here with the aim of helping to find the best modality of treatment for future patients with large gastric lipoma.

**Conclusion:**

We present, to the best of our knowledge, the first case of acute gastrointestinal bleeding from a gastric lipoma in the Czech Republic, discuss the existing literature on similar cases, and make recommendations for the treatment of this condition in line with the relevant ESGE guidelines.

## 1. Introduction

Gastrointestinal (GI) bleeding is a common acute condition, with approximately 400,000 cases requiring hospitalization annually in the United States. The incidence of GI bleeding worldwide, in the USA, Europe, and also in Asia is about 100 cases per 100,000 adults per year. More than 50% of these cases have their source in the upper GI tract, with upper GI tract tumors accounting for approximately 5%–7% of these cases. No exact data about the incidence of GI bleeding in the Czech Republic are available in the literature [1].

Subepithelial lesions (SELs) of the GI tract are mesenchymal in origin and can arise from various tissues. SELs smaller than 3 cm are generally considered benign, with common types including leiomyomas, schwannomas, granular cell tumors, heterotopic pancreatic tissue, lipomas, neurofibromas, and hemangiomas. SELs larger than 3–5 cm, those with mitotic counts greater than 2 per 10 high power fields, and those involving multiple layers are considered high-risk tumors for malignancy. Leiomyosarcomas, liposarcomas, Kaposi's sarcomas, GI stromal tumors, and metastases from tumors of other origins can rarely be identified in the GI tract [2].

Gastric lipomas represent only 1%–3% of all gastric tumors. They are generally asymptomatic and are often discovered incidentally during endoscopic or radiological examination. Rarely, gastric lipomas can cause bleeding due to mucosal erosion or ulceration, gastric outlet obstruction, or gastroduodenal intussusception [3, 4].

Lipomas exhibit characteristic endoscopic characteristics: they are yellow-colored and display the following signs: the “pillow” sign (an impression after compressing the tumor with forceps), the “tenting” sign (easy retraction of the overlying mucosa with biopsy forceps), and the “naked fat” sign (bite-on-bite forceps biopsy revealing lipocytes), which is pathognomonic of GI lipomas. While endoscopic mucosal biopsy is usually inconclusive, a fine needle biopsy under endoscopic ultrasound (EUS) control may provide a definitive diagnosis. EUS typically reveals a homogeneous, well-bordered, hyperechoic lesion arising from the submucosa. A CT scan shows a well-circumscribed submucosal mass with a uniform fat density, ranging from −70 to −120 Hounsfield units [5–8].

Gastric lipomas have a low risk of malignant transformation, which is why no endoscopic follow-up or resection is generally required. Only 13 cases of gastric liposarcoma had been described in the literature by the end of 2024, and malignant transformation from a lipoma has never been demonstrated. Microscopic blood losses or manifest symptomatic bleeding is a very rare complication in large gastric lipomas (> 4 cm). In a series of 32 patients with giant gastric lipomas, the main risk factor for bleeding was an ulcer overlying the lipoma. Ulceration can occur because of venous stasis, friction, and trauma of the lipoma against the contralateral gastric wall. Other symptoms of giant lipomas can include dyspepsia, abdominal discomfort, and gastric outlet obstruction [9–14].

## 2. Case Report

An 82-year-old man was admitted to the Department of Internal Medicine at the Military University Hospital in Prague due to COVID-19-associated pneumonia and melena. His main comorbidities were chronic lymphocytic leukemia (CLL), arterial hypertension, and mild chronic kidney disease. In the emergency room, the patient was stable on conventional oxygen therapy. Laboratory tests revealed severe anemia (hemoglobin level of 65 g/L), elevated leukocyte count (63.9 × 10^12^/L), increased urea (11.9 mmol/L) and creatinine level (129 μmol/L), and elevated C-reactive protein (CRP) level (76 mg/L). The patient received crystalloids, proton pump inhibitors, antibiotics, and two blood transfusions, all administered intravenously, along with oral antiviral therapy for COVID-19.

**Figure 1:**
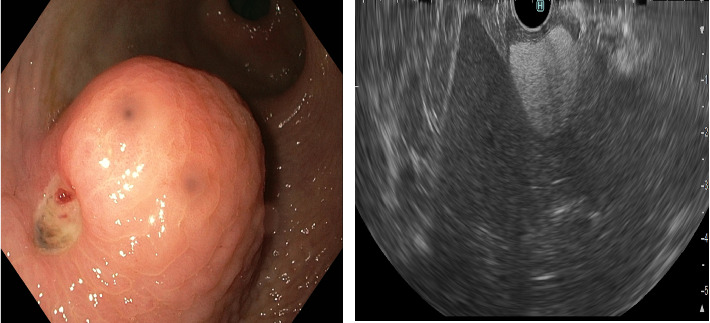
Endoscopic and EUS pictures of the lipoma: picture (a) showing large subepithelial lesion, ulceration, and visible vessel and picture (b) showing hyperechoic well-bordered tumor.

The following day, the patient underwent an upper GI endoscopy, which revealed a 3-4 cm SEL with a small ulceration and a visible vessel (Forrest IIa) in the antrum of the stomach. Immediate treatment with norepinephrine injection and bipolar coagulation was administered. The next day, an EUS was performed, describing the mass as a well-bordered, hyperechoic 32 × 25 mm tumor without signs of invasion, probably a lipoma (Figure 1). The treatment for the pneumonia and anemia was successful. Before discharge, considering the patient's age and comorbidities, a decision was made to schedule an endoscopic treatment of the lesion as a preferred alternative to surgery.

**Figure 2:**
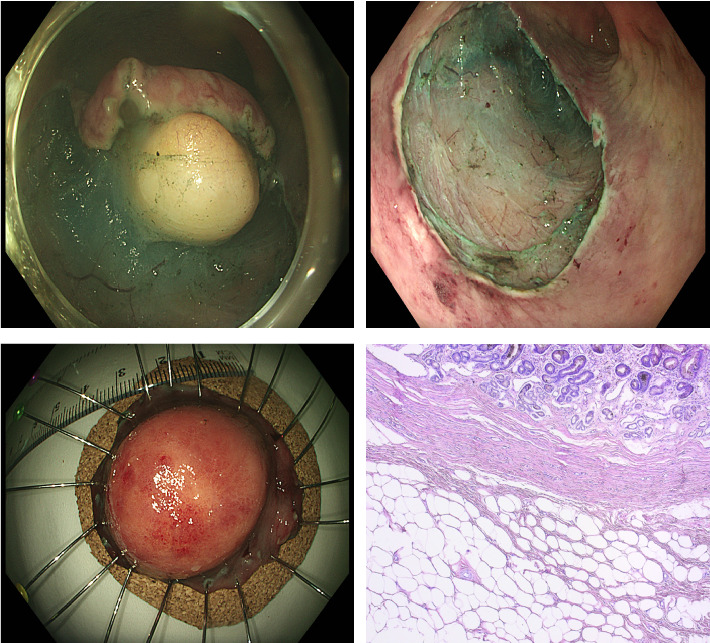
ESD of the lipoma and the histology finding: picture (a) showing the subepithelial lesion during the ESD and picture (b) showing the mucosa after resection. Picture (c) showing the removed lesion sent for histological evaluation and picture (d) showing the adipocytes.

Approximately 1 month later, the patient was readmitted for endoscopic treatment of the SEL. Upon admission, elevated leukocyte count and anemia (likely a relapse of CLL) were noted, with the need for another blood transfusion. The following day, a 45-minute endoscopic submucosal dissection (ESD) was performed, successfully removing the lesion, which was sent for histological evaluation. The diagnosis of an antral gastric lipoma was confirmed, with a tumor consisting of matured adipocytes without any atypical cells or lipoblasts (Figure 2). The procedure was performed without any complications, and the patient was discharged home after 2 days. After 1 month, there was a follow-up control of the patient at the outpatient care. With the result of the histology examination (benign lesion, successfully and completely removed by ESD), a follow-up endoscopy only in case of repeated anemia or bleeding was recommended.

## 3. Discussion

Lipomas are benign and predominantly asymptomatic tumors in the GI tract. They occur more often in men and individuals with obesity and type 2 diabetes mellitus. Lipomas are most commonly found in the colon, followed by the small intestine and the stomach, with only 5% of GI lipomas occurring in the stomach and less than 1% of gastric tumors being lipomas. Until 2021, only 217 cases of asymptomatic gastric lipomas had been reported in the literature. Histologically, these tumors are intramucosal mesenchymal tumors of mature adipocytes. Lipomas are typically asymptomatic and are often discovered incidentally during endoscopy, ultrasound, or CT scans [4, 5].

Upper GI bleeding caused by a large gastric lipoma is a very rare occurrence, with fewer than 50 case reports in the literature worldwide. Collecting these cases into a single study or meta-analysis is challenging due to the limited number of reports, which are primarily single case reports from endoscopy centers worldwide, reported retrospectively [5, 6].

Symptomatic lipomas are indicated for removal via surgical excision (partial gastrectomy or enucleation) or endoscopic resection. Kwang et al. reported a case series of 28 successful endoscopic resections of GI lipomas, including five in the stomach, without serious complications. Another study involving 15 patients with large GI lipomas > 2 cm, including three in the stomach, also reported successful endoscopic resection without perforation or bleeding. Endoscopic resection using the technique of ESD may be the first-line treatment for lipomas < 5 cm, with resection rates in the literature approaching 92% [6, 10, 15, 16].

In Table 1, we summarize 27 different cases of gastric lipoma presenting with acute GI bleeding reported in the literature since 1999. The mean age of the patients is 60 years (range 37–85), and the male-to-female ratio is 2.5. The most common location of the gastric lipoma is the antrum (22 patients). The mean size of the lesion is 6.66 cm. Twenty-two patients underwent surgical resection, while five patients underwent endoscopic resection.

Table 1 with the cases from previous years [6] has been supplemented with case reports from recent years.

Although the majority of gastric lipomas are asymptomatic, the ESGE guidelines emphasize the importance of a multidisciplinary approach to the treatment of all SELs. Both the patient's age and comorbidities and the size and localization of the lesion are very important for the treatment decision, which should be made by a multidisciplinary team (MDT) including a gastroenterologist, a surgeon, an oncologist, and an anesthesiologist. All symptomatic SELs should be treated, while even asymptomatic lesions, if large (> 4 cm), may carry a high risk of complications, such as bleeding or gastric outlet obstruction, especially in the antrum. Hence, it may be advisable to remove even asymptomatic SELs, in line with the ESGE guidelines [17].

EUS performed by an experienced endoscopist may inform the MDT's final decision, as it allows for more exact measurement of the size of the lesion, and the echogenicity of the SEL may also play a key role in determining the character of the lesion (lipomas are typically hyperechogenic). An EUS-navigated fine needle aspiration biopsy should be considered in cases involving SELs larger than 2 cm [17].

Nowadays, treatment options include the surgical approach (partial gastrectomy or enucleation) or an endoscopic resection (ESD). The decision as to which option to choose is dependent not only on the patient and case characteristics, as mentioned above, but also on the experience of the medical facility. In our case, the lipoma measured approximately 32 × 25 mm, which made endoscopic extraction feasible. After completing the ESD, the specimen was removed en bloc perorally with a standard retrieval net without fragmentation or complications. Where possible, mini-invasive endoscopy techniques are preferable, especially for older and comorbid patients. Alternative endoscopic methods to ESD, such as EMR, unroofing, or the loop-and-let-go technique, are described for gastric lipomas; however, these approaches may not ensure complete en bloc resection, particularly in larger lesions, and may limit histological evaluation. In our patient, the lesion was located in the antrum, measured over 3 cm, and was complicated by ulceration with active bleeding. Therefore, we considered ESD, in line with the ESGE guidelines, the safest and most appropriate technique, as it allowed precise submucosal dissection, en bloc removal, full histological assessment, and reliable hemostasis [17].

The ESGE does not recommend any follow-up in cases involving benign SELs. Following successful endoscopic treatment, there is no risk of the SEL progressing or causing other complications, such as a severe GI bleeding [17].

In the case of our 82-year-old patient, the submucosal lipoma measured 32 × 25 mm and was located in the antrum of the stomach, a common site for gastric lipomas that present with bleeding. The lipoma was identified during endoscopy after the patient presented with melena and severe anemia, likely exacerbated by his CLL and mild chronic kidney disease. These comorbidities complicated the patient's condition but also highlighted the importance of prompt diagnostic and mini-invasive interventions.

We examined the patient in the gastroenterology outpatient clinic 1 month after endoscopic treatment. He was without complications, without signs of recurrent bleeding, and his hemoglobin level was stable at 85 g/L. Given his dominant comorbidities and definitive benign histology, we referred the patient back to the hemato-oncology outpatient clinic.

We are presenting this case report because gastric lipomas are rare, and hence sharing successful diagnostic and therapeutic strategies is crucial. To the best of our knowledge, this is the first documented case of acute GI bleeding from a gastric lipoma in the Czech Republic. More case reports, studies, or even a European register of SELs could prove beneficial in improving treatment of this rare condition.

Our facility, a tertiary endoscopic center, is well equipped to handle these cases endoscopically. We have experienced endoscopists skilled in both the diagnosis and treatment of such conditions. In our patient's case, we accurately diagnosed the gastric lipoma through EUS and managed the initial active bleeding endoscopically. Our approach prioritizes endoscopic removal, preferably using ESD, whenever feasible. Following a multidisciplinary consensus, we successfully removed the risk-bearing lipoma using ESD, avoiding the need for more invasive surgical intervention.

We note that from Table 1, summarizing 27 cases of gastric lipoma presenting with acute GI bleeding reported since 1999, it is clear that a substantial number—22 out of 27 patients—underwent surgical resection of the stomach. The successful ESD performed on our patient highlights the effectiveness and safety of this minimally invasive technique, which aligns with ESGE guidelines and emerging literature that supports endoscopic resection as an effective alternative to surgery for managing symptomatic gastric lipomas.

## 4. Conclusion

Lipomas are benign GI tumors, and their localization in the stomach is quite rare. Bleeding (acute or microscopic) is a rare complication, with case reports to date indicating that it occurs more often in cases involving lipomas larger than 4 cm, especially those localized in the antrum with visible erosion or ulceration of the mucosa. ESD in a tertiary endoscopy center may currently be the optimal method of treatment, as demonstrated in our patient who successfully underwent this procedure rather than surgical resection. Our experience underscores the importance of advanced endoscopic skills and a multidisciplinary approach to effectively manage rare and challenging cases such as this.

## Figures and Tables

**Table 1:** 27 cases of gastric lipoma with acute gastrointestinal bleeding, their location, size, and treatment strategy.

Reference	Year	Location	Size	Shape	Treatment
Regge et al.	1999	Antrum	3.5 cm	Roundish + ulceration at the summit	Partial gastrectomy
Paksoy et al.	2005	Antrum	4 cm	Roundish + ulceration at the summit	Enucleation
Kibria et al.	2009	Greater curve	5 cm	Broad‐based polypoid lesion + 2 ulcerations	Surgical resection
Sadio et al.	2010	Fundus	4 cm	Roundish + ulceration at the summit	Partial gastrectomy
Ramdas et al.	2013	Junction body‐antrum	4 cm	Pedunculated	Surgical resection
Sagar et al.	2013	Antrum	8 cm	Ulceration	Surgical resection
Rao et al.	2013	Lesser curvature	12 cm	Normal mucosa	Surgical resection
Kumar et al.	2015	Antrum	NA	NA	Surgical resection
Almohsen et al.	2015	Antrum	8.5 cm	NA	Enucleation
Mao et al.	2015	Antrum	4.5 cm	Ulceration	Partial gastrectomy
Suarez et al.	2016	Antrum	NA	NA	Stepwise endoscopic snare resection
Krishnaraj et al.	2017	Antrum	8 cm	Bulge + ulceration at the summit	Enucleation
Termos et al.	2017	From the gastroesophageal junction to the pylorus along the lesser curvature	17 cm	Bulge + 4 cm linear ulceration	Enucleation
Koukias et al.	2017	Greater curvature	3 cm	NA	Hybrid endoscopic submucosal dissection
Cappel et al.	2017	Antrum	13 cm	NA	Partial gastrectomy
Cappel et al.	2017	Antrum	9 cm	NA	Partial gastrectomy
Yen et al.	2018	Antrum	4 cm	NA	Endoscopic resection
Sharayah et al.	2019	Antrum	5 cm	NA	Piecemeal resection
Han et al.	2019	Antrum	7 cm	NA	Endoscopic submucosal dissection
Sabbah et al.	2020	Antrum	4 cm	Ulceration at the summit	Partial gastrectomy
Rezaii et al.	2021	Antrum	4.5 cm	Partial obstruction of the lumen	Subtotal gastrectomy
Lalanda et al.	2021	Antrum	9 cm	Ulceration at the summit	Enucleation
Kumar et al.	2021	Antrum	5 cm	Ulceration	Laparoscopy resection
Margues et al.	2022	Antrum	6 cm	Ulceration at the summit	Subtotal gastrectomy
Jarrett et al.	2024	Antrum	4.5 cm	Ulceration	Laparoscopy resection
Jarrett et al.	2024	Antrum	9 cm	Ulceration	Surgical resection
Cherif et al.	2024	Antrum	5 cm	Ulceration	Distal gastrectomy

Abbreviation: NA = not available.

## Data Availability

The data that support the findings of this study are available on request from the corresponding author. The data are not publicly available due to privacy or ethical restrictions.
